# An Epidemiological Study of Anemia and Renal Dysfunction in Patients Admitted to ICUs across the United States

**DOI:** 10.1155/2012/938140

**Published:** 2012-08-14

**Authors:** Donald F. Brophy, Spencer E. Harpe, Daniel E. Carl, Gretchen M. Brophy

**Affiliations:** ^1^Department of Pharmacotherapy and Outcomes Science, VCU School of Pharmacy, P.O. Box 980533, Richmond, VA 23298-0533, USA; ^2^Division of Nephrology, VCU School of Medicine, Richmond, VA 23298, USA; ^3^Department of Epidemiology and Community Health, VCU School of Medicine, Richmond, VA 23298, USA; ^4^Department of Neurosurgery, VCU School of Medicine, Richmond, VA 23298, USA

## Abstract

The aims of this study were to determine the associations between anemia of critical illness, erythropoietin stimulating agents (ESA), packed red blood cell transfusions and varying degrees of renal dysfunction with mortality, and ICU- and hospital length of stay (LOS). This was a cross-sectional retrospective study of 5,314 ICU patients from USA hospitals. Hospital, patient demographics, and clinical characteristics were collected. Predictors of mortality and hospital and ICU LOS were evaluated using multivariate logistic regression models. The mean ICU admission hemoglobin in this study was 9.4 g/dL. The prevalence of ESA use was 13% and was associated with declining renal function; 26% of the ICU patients in this study received transfusion. ESA utilization was associated with 28% longer hospital LOS (*P* < 0.001). ICU LOS was increased by up to 18% in patients with eGFR rates of <30 and 30–59 mL/min/1.73 m^2^, respectively (*P* < 0.05) but not in those receiving dialysis. Mortality was significantly associated with renal dysfunction and dialysis with odds ratios of 1.94, 2.66 and 1.40 for the dialysis, and eGFR rates of <30 and 30–59 and mL/min/1.73 m^2^, respectively (*P* < 0.05). These data provide a snapshot of anemia treatment practices and outcomes in USA ICU patients with varying degrees of renal dysfunction.

## 1. Introduction

Anemia of critical illness (ACI) has been estimated to occur in up to 50% of patients admitted to the intensive care unit (ICU) [1–3]. Of further concern, following three days or longer in the ICU, nearly all patients will have developed anemia [1]. This often requires intervention, such as packed red blood cell (PRBC) transfusions or, until recently, pharmacological management with an erythropoietin stimulating agent (ESA). Both treatment options, while having varying degrees of efficacy in this population, carry substantial risks for morbidity and mortality [3–7].

The pathophysiology of ACI is complex and multi-factorial. Concurrent inflammation, renal dysfunction, blood loss from surgery, phlebotomy, and/or the gastrointestinal tract, coagulopathy, and bone marrow suppression are but a few of the many potential causes of ACI [8]. The clinical consequences of ACI are important; lack of adequate tissue oxygenation can contribute to cardiopulmonary failure, morbidity, mortality, and increased hospital length of stay (LOS).

 To date the major thrust of research has focused on identifying appropriate hemoglobin (Hb) treatment triggers and restrictive transfusion strategies and developing evidence-based guidelines for anemia management [9, 10]. While these are clearly important initiatives, they require well-designed, controlled clinical trials that may take years to complete. In the meantime, there remains a paucity of data regarding what happens in “real world” ICU environments across the United States.

To this end, we conducted a large epidemiological study of ACI across hospitals in the USA and evaluated patient and hospital demographics, renal function, ESA and PRBC usage, and clinical outcomes such as mortality, and ICU and hospital LOS.

## 2. Materials and Methods

This was a cross-sectional, population-based, retrospective study using the Solucient ACTracker database to determine the associations between ACI and renal dysfunction with three major outcome variables: mortality, and ICU and hospital LOS. The database contained approximately 10.5 million patient discharges from 550 hospitals nationwide. Thirty-three of the participating hospitals provided a complete set of laboratory data, drug utilization, hospital procedures, and patient outcomes data that were included in the final analysis. All data were deidentified to protect patient confidentiality. The study was approved by the Virginia Commonwealth University Institutional Review Board.

### 2.1. Study Population

Patients were included if they had an ICU admission of at least 24 hours between January 2001 and December 2005, were at least 18 years of age, had a hemoglobin (Hb) and serum creatinine (SCr) level obtained within 72 hours of hospital admission, or had a procedure code for dialysis (procedure code 39.95 (hemodialysis), 54.98 (peritoneal)). Exclusion criteria included patients with anemia of neoplastic diseases (285.22) or those receiving chemotherapy (V58.1). Patients with an admission Hb <12 g/dL were included in the final analysis and were grouped based on estimated glomerular filtration rate (eGFR) using the MDRD equation [11], which was categorized as >60, 30–59, <30 mL/min/1.73 m^2^, and those receiving dialysis, respectively.

### 2.2. Data Collection

The pertinent study data included hospital characteristics, patient demographics and disposition, severity of illness determined using Refined Diagnosis Related Group codes [12, 13], hospital and ICU LOS, ESA and PRBC transfusion usage based on prescriber orders, and admission Hb and SCr values.

### 2.3. Statistical Analyses

Descriptive statistics, including mean ± standard deviation (SD) and median (interquartile range), were used to characterize the central tendency and dispersion of continuous variables. Categorical variables were expressed as frequency and percentage within each category, and they were analyzed for significance by Pearson's chi-squared test. Differences in LOS were compared with respect to ESA and PRBC transfusion status with analysis of variance (ANOVA). There were 457 (2.4%) patients with missing ethnicity information in the database, and these patients were considered African-American for the purposes of calculating eGFR. There were 44 (0.23%) patients missing ICU LOS data, and these patients were excluded from the LOS analyses. To examine the effects of renal function status on hospital length of stay and mortality, regression analyses were conducted while controlling for potential confounding variables (including age, race, sex, ICU LOS, ESA use, transfusion status, mechanical ventilation or CPAP status, vasopressor use, severity of illness, and presence of following comorbid conditions: GI bleed, sepsis, and neurologic injury). Logistic regression was used for mortality estimates, and a generalized linear model with a normal distribution and a log link was used for the LOS analyses. In both cases, robust estimates of standard errors were calculated by clustering on hospitals. This was done to allow for a potential violation of independence among patients who were admitted to the same hospital. Data analysis was performed using Stata/SE version 11 (StataCorp LP, College Station, TX, USA) with a *P* value of <0.05 representing statistical significance.

## 3. Results

### 3.1. Hospital Characteristics

Thirty-three U.S. hospitals provided patient laboratory and clinical outcome data for this study (Table 1). The majority were classified as nonteaching hospitals with <200 beds. Nineteen percent of hospitals had >500 beds. The northeast USA had the most hospitals represented while the western USA had the least.

### 3.2. Patient Characteristics

The complete dataset contained 19,009 patients who had an ICU admission, SCr and Hb value. Of this total, 5,314 (28%) of these patients met the criteria for anemia (admission Hb < 12 g/dL).  Table 2 provides the population demographics. The mean age was 69.2 years however the age varied based on eGFR criteria. The majority of the study population was female and Caucasian. The mean baseline Hb was 9.4 g/dL for the study population, which was consistent across eGFR groups. Ninety-three percent of the population was directly admitted into an ICU environment (Table 3). Overall the majority of the study population admissions were classified as “Emergency” (53.6%) with the most frequent site of care being the medical ICU (66.4%). Only 376 (7%) of the population was not initially admitted to the ICU. The two most common severity of illness classifications for the population were “moderate” (33.5%) and “major” (35.1%); approximately 16% of the population was classified as “extreme” severity of illness. Seven hundred fifteen of 5,314 patients (13%) received ESA treatment while hospitalized. ESA usage was associated with declining eGFR and HD classification (*N* = 117 (5%), 118 (7%), 222 (22.2%), and 94 (47%) for each eGFR category and HD group, resp.). Comparatively, 1,398 (26%) patients received PRBC transfusion, and usage was consistent across eGFR (*N* = 526 (24%), 446 (27%), 238 (23.8%), and 26 (13%) for each eGFR and HD group, resp.).

### 3.3. Length of Stay and Mortality

Neither ESA nor PRBCs were associated with longer ICU LOS (Tables 4(a) and 4(b)). However, those classified in the eGFR categories of 30–59 and <30 mL/min/1.73 m^2^ had nearly 18 and 17% longer ICU LOS, respectively, compared to those with eGFR >60 mL/min/1.73 m^2^ (*P* < 0.005). Patients receiving ESAs had 28.4% longer hospital LOS (95% CI 14.6–43.9%, *P* < 0.001). Conversely, neither eGFR classification nor PRBC transfusion impacted total hospital LOS. Declining renal function and dialysis were the only variables significantly associated with increased mortality risk compared to those with eGFR >60 mL/min/1.73 m^2^ (Table 5).

## 4. Discussion

This study assessed anemia treatment practice patterns and associated clinical outcomes across a geographically diverse population of critically ill patients with varying degrees of renal dysfunction. While clinicians typically consider ACI a consequence of prolonged hospitalization, this study suggests that the prevalence of baseline anemia upon ICU admission is relatively common, approximately 28% of all ICU patients in this database study.

The study period ranged from 2001 to 2005, a time frame when ESA use was being actively evaluated in critically ill patients [4, 14, 15]. During this period, ESA use was proposed to reduce the number of PRBC transfusions and their associated morbidity. An interesting finding was that despite the relatively high baseline severity of illness and presence of mean Hb values of approximately 9.5 g/dL across all renal dysfunction groups, merely 13% of patients received ESA treatment. Low prevalence of ESA use in non-ICU, hospitalized patients has been shown previously [16]. Conversely, 26% received at least one PRBC transfusion, which was lower than previously published literature [2, 3, 14]. This is most likely explained by the mean baseline Hb of >9.0 g/dL across all groups. In many hospitals, the typical transfusion trigger is now 7 g/dL based on data by Hebert [17].

There were marked differences in the univariate and multivariate LOS results. The univariate results suggested that ESA usage was associated with longer ICU and hospital LOS; however when adjusted for patient severity and other factors, ESA usage was only associated with a 28% longer hospital LOS (Table 4(a)). Whether this was directly related to ESA use is impossible to know given the observational nature of this study. In fact the opposite could be argued in that the sicker patients with longer LOS were more likely to have an ESA intervention attempted. However, Corwin et al. previously found no increased ICU LOS associated with ESA utilization [4]. ESA use was not found to impact mortality in our study. However, previous subgroup analyses from prospective trials have suggested critically that ill trauma patients may have mortality benefit from recombinant erythropoietin [4, 14, 15]. Comparatively our study population contained merely 4% trauma surgery patients; therefore the dataset was not ideal to compare to previously published data.

This study found no association between PRBC utilization and increased LOS or mortality. In fact, there were few differences in LOS between those who received PRBCs and those who received no treatment. Previous prospective cohort studies have found a positive association between PRBC transfusion and LOS and mortality [2, 3, 5]. The disparate findings are likely the result of different study populations (e.g., less trauma in our study), differing levels of severity of illness, and differing degrees of anemia. A limitation of this dataset, unlike previously published trials, was that it did not quantify the number of PRBC transfusions patients received. Therefore if a “dose-response” existed between PRBC quantity, LOS, and mortality, we would not be able to identify this trend.

The presence of baseline renal dysfunction was associated with prolonged hospital and ICU LOS and mortality. While this has been known for some time, even mild renal dysfunction, 30–59 mL/min/1.73 m^2^, appeared to have a 40% increased mortality risk in this population. Interestingly there was no association between dialysis and increased LOS; however there were relatively few dialysis patients in the study population, therefore impacting the robustness of these data.

There are unavoidable limitations to this database study. A potential limitation is the external validity of these data to large, level 1 trauma centers. This database was comprised of primarily smaller, nonteaching institutions. This likely impacted the inclusion of more trauma patients as well as those with increased severity of illness. However, while there is a notion that there are differences between relatively smaller and larger teaching hospitals, there were no published data to support this notion. Similarly the geographic location of sites was more heavily weighted towards the northeast and southern USA. There is a possibility that anemia treatment practice patterns and transfusion thresholds differ across the USA. Third, database studies in general do not provide investigators with patient level data, such as ESA dose or PRBC units received, administration schedule, social history, or pertinent past medical history. Fourth, eGFR was used as a means of stratifying the anemia population. While it is debatable how accurately the eGFR estimates kidney function in critically ill patients, this served as a convenient means by which to stratify patient groups. Lastly, the potential for errors (e.g., clerical entry, hospital coding errors) in the database used for this study cannot be ignored.

## 5. Conclusion

In this cohort of anemic critically ill patients, the presence of baseline kidney dysfunction (eGFR < 60 mL/min/1.73 m^2^) upon ICU admission was associated with significantly prolonged ICU LOS and mortality. Twice as many patients received transfusion compared to ESA treatment. Blood transfusion was not associated with mortality or increased LOS. While ESA treatment was associated with a 28% longer hospitalization, these patients appeared to be more severely ill. Further prospective trials are needed to determine the best patient candidates for anemia treatment interventions in the ICU.

## Figures and Tables

**Table 1:** Hospital characteristics.

Parameter	*N* (%)
All hospitals	33 (100)
Teaching hospitals	5 (15)
Hospital beds	
<200	14 (42)
200–299	9 (27)
300–499	4 (12)
≥500	6 (19)
US geographic region	
Northeast	15 (46)
Midwest	6 (18)
South	10 (30)
West	2 (6)

**Table 2:** Baseline patient characteristics based on eGFR (mL/min/1.73 m^2^).

	Overall	≥60	30–59	<30	Dialysis
	(*n* = 5314)	(*n* = 2285)	(*n* = 1705)	(*n* = 1085)	(*n* = 239)
Mean age, y (SD)	69.2 (15.0)	65.1 (16.5)	73.6 (12.0)*	72.0 (13.4)*	64.5 (15.8)
Gender, female, *n* (%)	2861 (53.8)	1179 (51.6)	959 (56.3)*	609 (56.1)*	114 (47.7)*
Ethnicity, *n* (%)					
White	4394 (82.7)	1874 (82.0)	1473 (86.4)	889 (81.9)	158 (66.1)
Black	729 (13.7)	334 (14.6)	182 (10.7)	147 (13.6)	66 (27.6)
Other	191 (3.6)	77 (3.4)	50 (2.9)	49 (4.5)	15 (6.3)
Mean Hb, g/dL (SD)	9.4 (1.2)	9.5 (1.4)	9.4 (1.3)	9.2 (1.4)*	9.4 (1.2)
Mean eGFR, (SD)	59.0 (39.9)	93.6 (35.5)	45.1 (8.7)*	18.4 (7.2)*	N/A
Mean SCr mg/dL (SD)	1.9 (1.9)	0.8 (0.2)	1.5 (0.3)*	3.8 (2.2)*	6.2 (3.1)*

*P < 0.001 compared to eGFR > 60 mL/min/1.73 m^2^.

**Table 3:** Admission characteristics based on eGFR (mL/min/1.73 m^2^).

	Overall	≥60	30–59	<30	Dialysis	*P*
	(*n* = 5314)	(*n* = 2285)	(*n* = 1705)	(*n* = 1085)	(*n* = 239)	
Admission type, *n* (%)						<0.001
Elective/routine	971 (18.3)	563 (24.6)	285 (16.7)	96 (8.9)	27 (11.3)	
Emergency	2849 (53.6)	1047 (45.8)	988 (57.9)	669 (61.7)	145 (60.7)	
Urgent	988 (18.6)	429 (18.8)	281 (16.5)	224 (20.7)	54 (22.6)	
Unknown	506 (9.5)	246 (10.8)	151 (8.9)	96 (8.9)	13 (5.4)	
Admission, *n* (%)						
ICU	**4934 (93)**					<0.001
Medical	3277 (66.4)	1245 (58.3)	1090 (68.7)	781 (78.3)	161 (75.2)	
Trauma surgery	196 (4.0)	121 (5.7)	61 (3.8)	9 (0.9)	5 (2.3)	
Nontrauma surgery	1461 (29.6)	770 (35.1)	436 (27.5)	207 (20.8)	48 (22.4)	
Non-ICU	**376 (7)**					0.462
Medical	205 (54.5)	74 (50.3)	62 (53.5)	55 (62.5)	14 (56.0)	
Trauma surgery	17 (4.5)	8 (5.4)	7 (6.0)	2 (2.3)	0	
Non-trauma surgery	154 (41.0)	65 (44.2)	47 (40.5)	31 (35.2)	11 (44.0)	
Severity of Illness, *n* (%)						<0.001
Minor	834 (15.7)	405 (17.7)	234 (13.7)	178 (16.4)	17 (7.1)	
Moderate	1782 (33.5)	659 (28.8)	614 (36.0)	406 (37.4)	103 (43.1)	
Major	1866 (35.1)	855 (37.4)	568 (33.3)	355 (32.7)	88 (36.8)	
Extreme	832 (15.7)	366 (16.0)	289 (16.9)	146 (13.5)	31 (12.9)	

**Table:** (a) Association between eGFR category, ESA use, and transfusion status in hospital LOS

	Unadjusted data		Adjusted data*	
	Relative change (95% CI)	*P*	Relative change (95% CI)	*P*
eGFR range				
Dialysis	16.6% (−0.03–40.0%)	0.095	0.06% (−0.12–28.7%)	0.537
<30	0.02% (−0.07–12.9%)	0.620	0.02% (−0.12–19.7%)	0.763
30–59	−0.01% (−0.08–6.8%)	0.837	0.01% (−0.10–14.5%)	0.819
≥60	—	—	—	—
ESA use	94.9% (74.9–117.1%)	<0.001	28.4% (14.6–43.9%)	<0.001
PRBC	−0.11% (−0.20–0.01%)	0.028	0.01% (−0.07–8.9%)	0.914

*Adjusted for mortality, age, race, sex, ICU LOS, anemia status, ESA use, transfusion status, mechanical ventilation status, CPAP status, vasopressor use, severity of illness, and presence of, following comorbid conditions, GI bleed, sepsis, acute renal failure, and neurologic injury.

**Table:** (b) Association between eGFR category, ESA use, and transfusion status in ICU LOS

	Unadjusted data		Adjusted data*	
	Relative change (95% CI)	*P*	Relative change (95% CI)	*P*
eGFR range				
Dialysis	0.06% (−0.22–13.3%)	0.505	0.04% (−0.17–29.0%)	0.757
<30	0.03% (−0.06–12.5%)	0.535	17.9% (0.07–29.7%)	0.001
30–59	0.04% (−0.04–12.5%)	0.379	16.8% (0.05–30.1%)	0.005
≥60	—	—	—	—
ESA use	96.3% (71.7–124.4%)	<0.001	0.04% (−0.07–15.8%)	0.479
PRBC	0.16% (−0.26–0.04%)	0.008	0.01% (−0.08–10.9%)	0.855

*Adjusted for mortality, age, race, sex, ICU LOS, ESA use, transfusion status, mechanical ventilation status, CPAP status, vasopressor use, severity of illness, and presence of, following comorbid conditions, GI bleed, sepsis, acute renal failure, and neurologic injury.

**Table 5:** Odds ratios for mortality based on eGFR, ESA, and PRBC treatment.

	Unadjusted data		Adjusted data*	
	OR (95% CI)	*P*	OR (95% CI)	*P*
eGFR (mL/min/1.73 m^2^)				
Dialysis	1.4 (0.87–2.20)	0.164	1.94 (1.22–3.08)	0.005
<30	3.1 (2.46–3.89)	<0.001	2.66 (2.05–3.45)	<0.001
30–59	1.6 (1.37–1.92)	<0.001	1.40 (1.20–1.66)	<0.001
≥60	—	—	—	—
ESA treatment	1.1 (0.79–1.53)	0.587	0.86 (0.43 –1.72)	0.674
PRBC treatment	0.91 (0.67–1.25)	0.571	1.10 (0.82 –1.38)	0.641

*Adjusted for age, race, sex, ICU LOS, ESA use, transfusion status, mechanical ventilation or CPAP status, vasopressor use, severity of, illness, and presence of, following comorbid conditions, GI bleed, sepsis, and neurologic injury.
